# Peptone‐mediated glucagon‐like peptide‐1 secretion depends on intestinal absorption and activation of basolaterally located Calcium‐Sensing Receptors

**DOI:** 10.14814/phy2.14056

**Published:** 2019-04-24

**Authors:** Ida M. Modvig, Rune E. Kuhre, Jens Juul Holst

**Affiliations:** ^1^ Department of Biomedical Sciences NNF Center for Basic Metabolic Research Faculty of Health and Medical Sciences University of Copenhagen Copenhagen Denmark

**Keywords:** Amino acid sensing, Calcium‐Sensing Receptor, glucagon‐like peptide 1, peptide transporter 1, peptone

## Abstract

Protein intake robustly stimulates the secretion of the incretin hormone, glucagon‐like peptide‐1 (GLP‐1) but the molecular mechanisms involved are not well understood. In particular, it is unknown whether proteins stimulate secretion by activation of luminal or basolateral sensors. We characterized the mechanisms using a physiologically relevant model – the isolated perfused proximal rat small intestine. Intraluminal protein hydrolysates derived from meat (peptone; 50 mg/mL) increased GLP‐1 secretion 2.3‐fold (from a basal secretion of 110 ± 28 fmol/min). The sensory mechanisms underlying the response depended on di/tripeptide uptake through Peptide Transporter 1 (PepT1) and subsequent basolateral activation of the amino acid sensing receptor, Calcium‐Sensing Receptor (CaSR), since inhibition of PepT1 as well as CaSR both attenuated the peptone‐induced GLP‐1 response. Supporting this, intraluminal peptones were absorbed efficiently by the perfused intestine (resulting in increased amino acid concentrations in the venous effluent) and infusion of amino acids robustly stimulated GLP‐1 secretion. Inhibitors of voltage‐gated L‐type Ca^2+^ channels had no effect on secretion suggesting that peptone‐mediated GLP‐1 secretion is not mediated by L‐cell depolarization with subsequent opening of these channels. Specific targeting of CaSR could serve as a target to stimulate the endogenous secretion of GLP‐1.

## Introduction

The incretin hormone, glucagon‐like peptide‐1 (GLP‐1) is an important regulator of glucose homeostasis as it strongly potentiates glucose‐induced insulin secretion, inhibits gastric emptying, and lowers food intake (Baggio and Drucker 2007; Holst 2007). Because of these effects, GLP‐1 is an attractive target for the treatment of both type 2 diabetes and obesity, and GLP‐1‐based drugs are now used for both indications (Zander et al. 2002; Drucker and Nauck 2006; Pi‐Sunyer et al. 2015). An alternative approach for targeting the GLP‐1 axis for treatment of type 2 diabetes and obesity, which has gained increasing interest and recognition, is to selectively activate the secretory machinery of the GLP‐1‐secreting L cell. This strategy may prove to be superior to GLP‐1 receptor agonists or DPP‐4 inhibitors for two reasons: (1) Part of the insulinotropic and appetite‐inhibiting effect of GLP‐1 is thought to be caused by the activation of vagal afferents in the intestinal mucosa (immediately after its release), and this pathway may not be accessible for the GLP‐1 enhancers or analogues (Furness et al. 1999; Holst and Deacon 2005; Steinert 2011), (2) Targeting the L‐cell secretion may also, at least to some extent, lead to increased secretion of colocalizing hormones including peptide YY (PYY), neurotensin, oxyntomodulin, and glucose‐dependent insulinotropic polypeptide (GIP), which inhibit appetite and/or are insulinotropic (Svendsen et al. 2015). Targeting L‐cell secretion, however, requires detailed knowledge of the molecular mechanisms that trigger secretion and although progress has been made, many details are still missing.

Ingested proteins are among the most powerful stimuli for GLP‐1 secretion in humans (Elliott et al. 1993; Hall et al. 2003; Raben et al. 2003; van der Klaauw et al. 2013) but the sensing mechanisms are not well characterized. In vivo and in vitro studies have pointed to a role for Peptide Transporter 1 (PepT1), a proton‐coupled di/tripeptide transporter, in peptide‐triggered GLP‐1 secretion (Matsumura et al. 2005; Diakogiannaki et al. 2013; Dranse et al. 2018). A mechanism involving the H^+^‐coupling of PepT1 transport, leading to membrane depolarization (indirectly through sodium, as the protons instantly will be transported out of the cell again through a Na^+^/H^+^ exchanger) and calcium influx through L‐type voltage‐gated calcium channels has been proposed (Matsumura et al. 2005). In addition, several amino acids (the final digestive product of proteins) have been demonstrated to stimulate GLP‐1 secretion in various experimental models (Herrmann et al. 1995; Reimann et al. 2004; Greenfield et al. 2009; Tolhurst et al. 2011; Pais et al. 2016); however, our understanding of the underlying molecular pathways is generally incomplete but suggested mechanisms include activation of amino acid sensing G‐protein‐coupled receptors (Calcium‐Sensing Receptor (CaSR) and Umami Taste Receptor (T1R1/T1R3), GPRC6A) (Mace et al. 2012; Diakogiannaki et al. 2013; Pais et al. 2016). Most importantly, it is unknown whether amino acids or di‐ and tripeptides stimulate secretion before, during or after intestinal absorption, or in other words, whether secretion is caused by activation of luminal, intracellular, or basolateral sensors. Given that protein digestion generates a mixture of breakdown products, ranging from large oligopeptides to smaller di/tripeptides and individual amino acids, the ability to sense protein digestion products is likely to involve several stimulation pathways. To characterize the molecular mechanisms responsible for protein‐stimulated GLP‐1 secretion, we performed a series of experiments on isolated perfused rat small intestine, stimulating the preparation with intraluminal peptones while key candidate sensors/transporters were activated or inhibited by selective compounds.

## Materials and Methods

### Ethical considerations

Studies were carried out with permission from the Danish Animal Experiments Inspectorate (2018‐15‐0201‐01397) and the local ethics committee (EMED, P18‐336). All experiments were performed in accordance with the guidelines of Danish legislation governing animal experimentation (1987) and the National Institutes of Health.

### Animals

Male Wistar rats (200–250 g) were obtained from Janvier (Le Genest‐Saint‐Isle, France), and housed two per cage and kept on a 12‐h light/dark cycle with free access to water and standard chow. Rats were acclimatized for at least a week.

### Perfusion of the proximal small intestine

At the day of experiment, nonfasted rats were anesthetized with a subcutaneous injection of Hypnorm/Midazolam and placed on a 37°C heated operating plate. When lack of reflexes was established, the operation was started by opening of the abdominal cavity. The vascular supply to colon was ligated and the colon was carefully removed. The small intestine was divided into its proximal and distal half. The vascular supply to the distal half was ligated so that only the proximal half was perfused (≈35 cm, CV = 13.6%). A plastic tube was placed in the lumen of the proximal jejunum and the intestinal contents were carefully removed by flushing with prewarmed (37°C) isotonic saline. Throughout the experimental protocol, a constant luminal flow of heated saline (37°C) was applied (0.25 mL/min) via a syringe pump. A catheter was inserted into the superior mesenteric artery and the intestine was vascularly perfused with heated perfusion buffer (37°C) at a constant flow rate of 7.5 mL/min. A metal catheter was inserted into vena portae to collect the venous effluent. Rats were euthanized by perforation of the diaphragm as soon as proper flow was apparent. As an indicator of the health of the intestine, perfusion pressure was continuously monitored. To allow for equilibration of the system, the intestine was perfused for 25 min before initiation of the experimental protocol. The venous effluent was collected for 1 min periods via the draining catheter using a fraction collector. The samples were immediately chilled on ice and stored at −20°C until analysis. The procedure is described in more detail elsewhere (Kuhre et al. 2015).

### Perfusion protocol and test substances

Each protocol started with a 10‐min baseline period of luminal saline (0.9% NaCl) infusion followed by addition of various test substances to either the luminal or arterial side of the preparation. Vascular infusion of test substances was carried out (at an infusion rate of 0.375 mL/min) for 5–15 min periods (indicated in the results section). Luminally applied test substances were administered for 15‐min periods at an initial rate of 0.5 mL/min for the first 2 min (to replace saline solution in the lumen) followed by 0.25 mL/min throughout the remaining stimulation period. Immediately after each stimulation period, the lumen was flushed with a similar bolus of isotonic saline (0.5 mL/min for 2 min), followed by infusion at a flow rate of 0.25 mL/min (baseline). Bombesin (10 nmol/L; BBS), a known GLP‐1 secretagogue, was infused at the end of each experiment as a positive control for hormone responses.

All test substances were purchased from Sigma‐Aldrich (Brøndby, Denmark): peptone, cat. no. P5905; glycyl‐sarcosine, cat. no. G3127; nifedipine, cat. no. N7634; 4‐(aminomethyl)benzoic acid, cat. no. 283746; calindol hydrochloride, cat. no. SML1386; bombesin, B4272; and NPS2143 hydrochloride, cat. no. SML0362. Test stimuli applied to the luminal side of the gut were diluted in isotonic saline, whereas compounds applied to the vascular side were diluted in perfusion buffer (PB). PB was a Krebs‐Ringer bicarbonate buffer supplemented with 0.1% (w/v) bovine serum albumin (cat. no. 1.12018.0500, Merck Ballerup, Denmark), 5% (w/v) dextran T‐70 (Pharmacosmos, Holbaek, Denmark), 3.5 mmol/L glucose, and 5 mmol/L of each pyruvate, fumarate, and glutamate (Sigma Aldrich, Broendby, Denmark). Prior to every experiment, a mixture of amino acids, Vamin^®^ (cat. no.11338, Fresenius‐kabi, Uppsala Sweden), was added (2 mL/L) to the PB along with 10 *μ*mol/L 3‐isobutyl‐1‐methylxanthine (IBMX) (cat no. 5879, Sigma Aldrich). The PB was filtered (at 0.25 *μ*m) to remove potential particles/precipitates and the pH was adjusted to 7.4–7.5. Before entering the organ, the PB was heated to 37°C and gassed with a mixture of 95% O_2_ and 5% CO_2_ to maximally increase the oxygen concentration and ensure a pH of 7.4.

### Hormone and L‐amino acid analysis

GLP‐1 concentrations in venous effluents were measured by an in house radioimmunoassay. An antiserum directed against the amidated C‐terminus of GLP‐1 (code no. 89390) was used, allowing measurement of total amidated GLP‐1. I^125^‐labeled GLP‐1 7‐36NH_2_ (a gift from Novo Nordisk A/S, Bagsværd, Denmark) was used as a tracer. Hormone concentrations were calculated by interpolation to a GLP‐1 standard curve set up in perfusion buffer using synthetic GLP‐1 7‐36NH2 (H‐6795‐GMP; Bachem). The experimental detection limit was 1 pmol/L and the coefficient of variation was <6% at 20 pmol/L.

Amino acid concentrations in venous effluents were measured using a colorimetric L‐amino acid assay kit from Abcam (cat. no. ab65347, Cambridge, UK), following the provided protocol. Reportedly, the assay detects all L‐amino acids but glycine with a sensitivity of 40 *μ*mol/L.

### Quantification and statistical analysis

Hormone outputs were calculated by multiplying perfusion flow (7.5 mL/min) with the GLP‐1 concentration in the effluent (measured in pmol/L). Hormone outputs are presented as mean values ± SEM. Statistical calculations were performed using GraphPad Prism 7 software (La Jolla, CA, USA). To test for statistical significance of responses, GLP‐1 outputs within the test period (10 min) were compared after subtraction of baseline GLP‐1 outputs (calculated over 10 min preceding test substance administration). Student's *t*‐test and one‐way ANOVA for repeated measurements followed by Bonferroni post hoc test were used as indicated in the results section. *P*‐values <0.05 were considered statistically significant.

## Results

### Luminal peptone stimulates GLP‐1 secretion from the proximal small intestine

**Figure 1:**
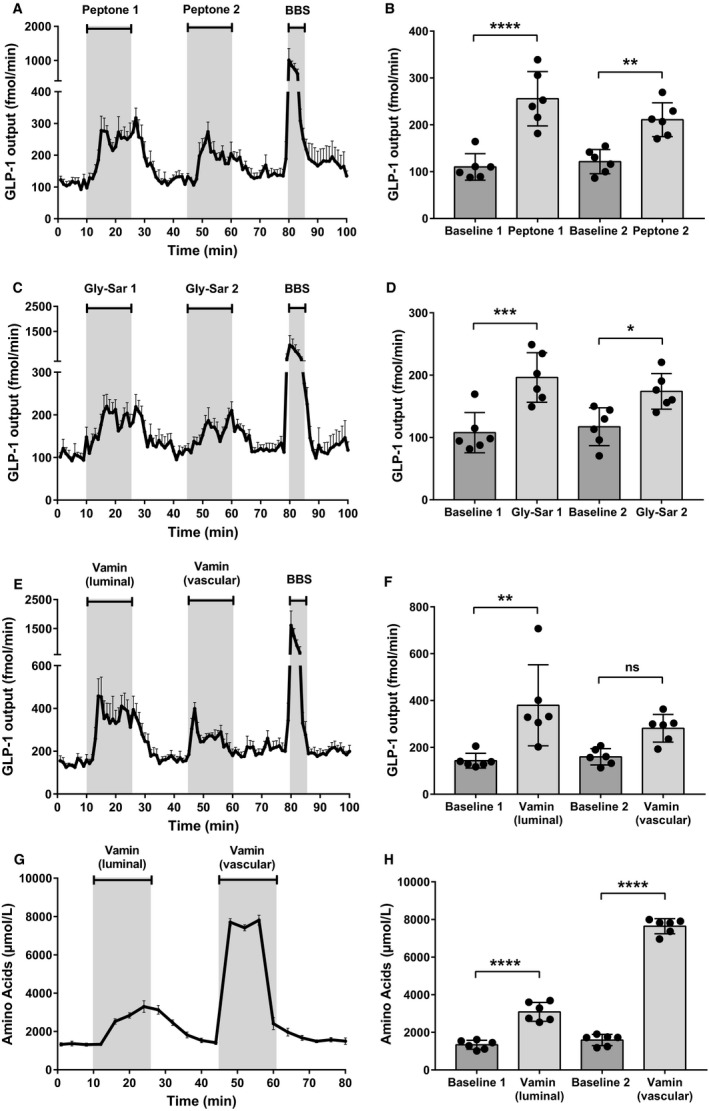
Luminal peptone stimulates GLP‐1 secretion from the proximal small intestine. (A) Total GLP‐1 outputs shown as means ± SEM. Peptone (50 mg/mL) was infused intraluminally between minute 11–25 and minute 46–60. Bombesin (BBS; 10 nmol/L), a positive control, was infused between minute 80–85. (B) Mean GLP‐1 outputs at baseline (Baseline 1 and 2) and following stimulation with luminal peptones (Peptone 1 and 2). (C) Total GLP‐1 outputs shown as means ± SEM. Gly‐Sar (22 mg/mL) was infused intraluminally between minute 11–25 and minute 46–60 (D) Mean GLP‐1 outputs at baseline (baseline 1 and 2) and following stimulation with luminal Gly‐Sar (Gly‐Sar 1 and 2). (E) Total GLP‐1 outputs shown as means ± SEM. Vamin (51 mg/mL) was infused intraluminally between minute 11–25. Vamin (4.25 mg/mL) was infused intravascularly between minute 46–60. (F) Mean GLP‐1 outputs at baseline (baseline 1 and 2) and following stimulation with luminal and vascular Vamin, respectively. (G) Total amino acid concentrations in venous effluent shown as means ± SEM. (H) Mean concentrations of total amino acids at baseline (baseline 1 and baseline 2) and following stimulation with, respectively, luminally and vascularly administered Vamin. Statistical significance was tested by one‐way ANOVA for repeated measurements followed by Bonferroni post hoc test; NS: nonsignificant: *P* > 0.05. *P < 0.05, **P < 0.01, ***P < 0.001, ****P < 0.0001. *n* = 6 for all groups.

Luminal infusions of peptone derived from meat (50 mg/mL) stimulated GLP‐1 secretion in double administration control experiments (Fig. 1A). Both infusions (P1 and P2) increased GLP‐1 secretion approximately twofold compared to baseline GLP‐1 secretion (B1 and B2) (B1: 110 ± 28 fmol/min vs. P1: 256 ± 58 fmol/min, *P* < 0.05, B2: 122 ± 26 fmol/min vs. P2: 211 ± 36 fmol/min, *P* < 0.05, *n* = 6) (Fig. 1B).

Since peptone is a mixture of enzymatically generated peptide fragments, both free amino acids and oligopeptides are present in the peptone mixture. To separate the relative contributions of free amino acids and di/tripeptides, we stimulated the intestines in separate experiments with a dipeptide resistant to luminal degradation; Glycyl‐Sarcosine (Gly‐Sar; 22 mg/mL), as well as a mixture of 18 different amino acids (not including glutamine), Vamin^®^, administered intraluminally as well as intra‐arterially (Fig. 1C+1E). Luminally infused Gly‐Sar (G1) increased GLP‐1 secretion almost twofold compared to baseline (B1) (B1: 108 ± 32 fmol/min vs. G1: 196 ± 40 fmol/min, *P* < 0.05, *n* = 6) (Fig. 1D). In addition, both luminally (V1) and vascularly (V2) infused amino acids (51 and 4.25 mg/mL, respectively) increased GLP‐1 secretion with luminal amino acids increasing GLP‐1 levels 2.6‐fold compared to baseline secretion (B1) (B1: 143 ± 32 fmol/min vs. V1: 380 ± 173 fmol/min, *P* < 0.05, B2: 160 ± 35 fmol/min vs. V2: 282 ± 59 fmol/min, *P* = 0.2235, *n = *6) (Fig. 1F). This indicates that both free amino acids and dipeptides may contribute to the peptone‐induced GLP‐1 release.

Since the GLP‐1 response to luminal amino acids may be secondary to amino acid absorption, we measured the concentration of amino acids in the venous effluent before and after stimulation with luminal‐infused amino acids (Fig. 1G). The luminal stimulus increased the total amino acid concentration in the venous effluent 2.3‐fold compared to baseline (B1_aa_: 1336 ± 239 *μ*mol/L vs. V1_aa_: 3088 ± 501 *μ*mol/L, *P* < 0.05, *n* = 6) (Fig. 1H), showing that luminal amino acids are rapidly absorbed and transported to the vascular side of the gut.

### Blockage of PepT1 inhibits peptone‐stimulated GLP‐1 secretion and peptide absorption

**Figure 2:**
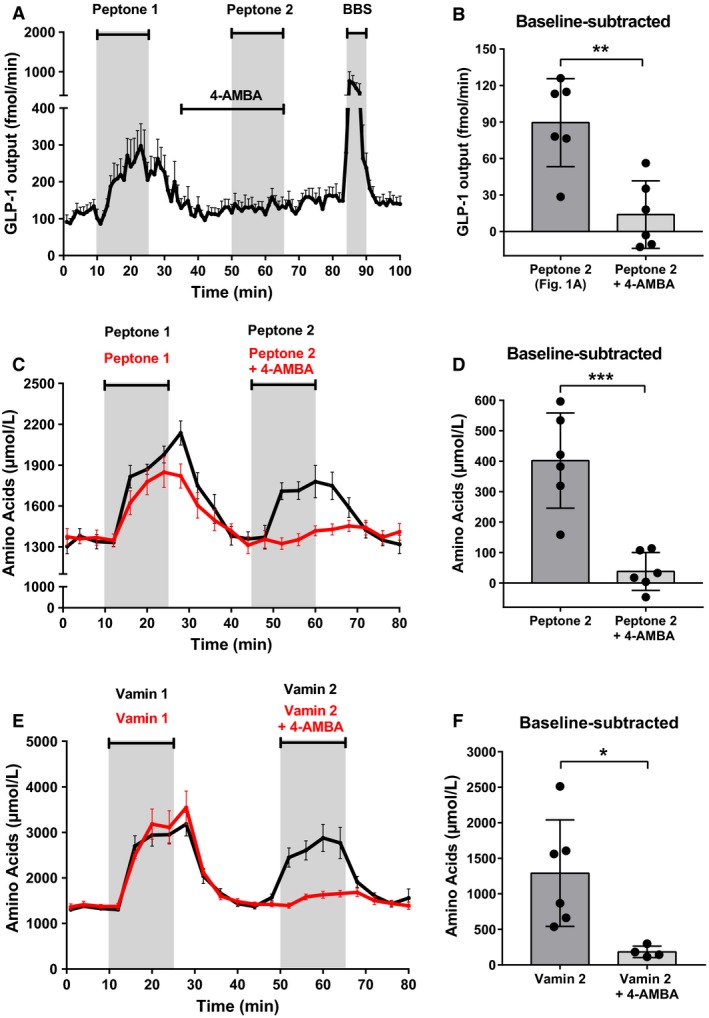
Blockage of PepT1 inhibits peptone‐induced GLP‐1 secretion and peptide absorption. (A) Total GLP‐1 outputs shown as means ± SEM. Peptone (50 mg/mL) was intraluminally infused between minute 11–25 and minute 51–65. A PepT1 blocker, 4‐AMBA (20 mmol/L), was intraluminally infused between min 36–65. (B) Mean baseline‐subtracted GLP‐1 outputs in response to peptone stimulation with and without 4‐AMBA (Peptone 2; Fig. 1 vs. Peptone 2 + 4‐AMBA). (C) Total amino acid concentrations in venous effluent shown as means ± SEM. Peptone (50 mg/mL) was intraluminally infused between minute 11–25 and minute 46–65. A PepT1 blocker, 4‐AMBA (20 mmol/L), was intraluminally infused between min 36–65. (D) Mean amino acid concentrations subtracted baseline during peptone stimulation with and without 4‐AMBA (Peptone 2 vs. Peptone 2 + 4‐AMBA). (E) Total amino acid concentrations in venous effluent shown as means ± SEM. Vamin (51 mg/mL) was intraluminally infused between minute 11–25 and minute 46–65. A PepT1 blocker, 4‐AMBA (20 mmol/L), was intraluminally infused between min 36–65. (F) Mean amino acid concentrations (after subtraction of baseline values) during Vamin stimulation with and without 4‐AMBA (Vamin 2 vs. Vamin 2 + 4‐AMBA). Statistical significance was tested by an unpaired student's *t*‐test; NS: nonsignificant: *P* > 0.05. *P < 0.05, **P < 0.01, ***P < 0.001, ****P < 0.0001. *n* = 6 for all groups except Vamin + 4‐AMBA (Fig. 2E), *n* = 4.

To study whether absorption of peptides via PepT1 is important for peptone‐stimulated GLP‐1 secretion, we blocked PepT1‐mediated uptake with the nontranslocated competitive inhibitor, 4‐aminomethyl benzoic acid (4‐AMBA; 20 mmol/L), while stimulating the intestine with luminal peptones. This resulted in elimination of the peptone‐mediated GLP‐1 response (Fig. 2A, *n* = 6), demonstrating that uptake via PepT1 is essential for the peptone‐mediated GLP‐1 secretion. When subtracting baselines, GLP‐1 secretion in response to the second peptone stimulation was much lower after 4‐AMBA administration, compared to the response to the second peptone stimulation in control experiments (P2: Fig. 1A; subtracted baseline) (P2: 90 ± 36 fmol/min vs. P2_4‐AMBA_: 14 ± 28 fmol/min, *P* < 0.05, *n* = 6) (Fig. 2B).

To test whether peptides absorbed by PepT1 are degraded to amino acids and transported to the vascular supply in our experimental model, we measured the total amino acid concentration in the venous effluent following stimulation with luminal peptones. As expected, the concentration of (total) amino acids increased robustly and quickly following peptone administration (B1_aa_: 1340 ± 122 *μ*mol/L vs. P1_aa_: 1995 ± 129 *μ*mol/L, *P* < 0.05, *n* = 6, B2_aa_: 1370 ± 163 *μ*mol/L vs. P2_aa_: 1772 ± 201 *μ*mol/L, *P* < 0.05, *n* = 6) (Fig. 2C; black curve). However, since the increase in amino acids in the venous effluent could arise from the uptake of free amino acids present in the peptone mixture, rather than from absorption of di‐ and tripeptides, we next blocked PepT1 and administered intraluminal peptones (Fig. 2C; red curve). In these experiments, 4‐AMBA abolished the rise in vascular amino acids during the second peptone stimulation (P2_aa_) (baseline‐subtracted values; P2_aa_: 402 ± 156 *μ*mol/L vs. P2_aa+4‐AMBA_: 38 ± 62 *μ*mol/L, *P* < 0.05, *n* = 6) (Fig. 2D), demonstrating that PepT1 is crucial for the absorption of di‐ and tripeptides.

However, since the peptone‐induced increase in vascular amino acids derived almost completely from the uptake of di/tripeptides, as opposed to the uptake of free amino acids present in the peptone mixture (Fig. 2C+D), the specificity of 4‐AMBA as a PepT1 blocker was tested in control experiments, where amino acids (Vamin; 51 mg/mL) were infused intraluminally, while PepT1 was blocked with 4‐AMBA (20 mmol/L) (Fig. 2E; red curve). The amount of amino acids in the venous effluent was measured (V2_aa+4‐AMBA_; Fig. 2F) and compared to a Vamin double administration control experiment (Fig. 2E; black curve) (V2_aa_; Fig. 2F). In these experiments, 4‐AMBA inhibited the rise in vascular amino acids during luminal Vamin stimulation (baseline‐subtracted values; V2_aa_: 1291 ± 749 *μ*mol/L vs. V2_aa+4‐AMBA_: 184 ± 81 *μ*mol/L, *P* < 0.05, *n* = 4–6*,* Fig. 2F), demonstrating that 4‐AMBA is probably not fully specific for PepT1, but also blocks the uptake of free amino acids present in the intestinal lumen.

### L‐type Ca^2+^‐channels are not involved in peptone‐stimulated GLP‐1 secretion

**Figure 3:**
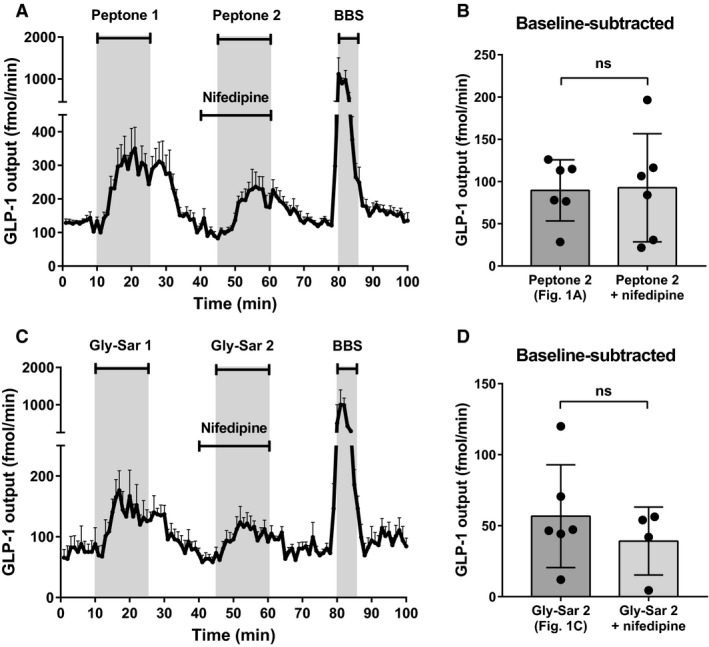
L‐type Ca^2+^‐channels are not involved in peptone‐stimulated GLP‐1 secretion. (A) Total GLP‐1 outputs shown as means ± SEM. Peptone (50 mg/mL) was intraluminally infused between minute 11–25 and minute 46–60. Nifedipine (10 *μ*mol/L), an L‐type Ca_v_^2+^‐channel inhibitor, was infused intravascularly between minute 40–60. (B) Mean values of the baseline‐subtracted peptone‐stimulated GLP‐1 outputs with and without nifedipine (Peptone 2; Fig. 1 vs. Peptone 2 + nifedipine). (C) Total GLP‐1 outputs shown as means ± SEM. Gly‐Sar (22 mg/mL) was intraluminally infused between minute 11–25 and minute 46–60. Nifedipine (10 *μ*mol/L) was infused intravascularly between minute 40–60. (D) Mean values of the baseline‐subtracted Gly‐Sar‐stimulated GLP‐1 outputs with and without nifedipine (Gly‐Sar 2; Fig. 1 vs. Gly‐Sar 2 + nifedipine). Statistical significance was tested by an unpaired student's *t*‐test; NS: nonsignificant: *P* > 0.05. *P < 0.05, **P < 0.01, ***P < 0.001, ****P < 0.0001. *n* = 6 for all groups except Gly‐Sar + nifedipine, *n* = 4.

To examine whether the mechanism of peptone‐mediated secretion involved depolarization of the L cells and opening of voltage‐gated (L‐type) Ca^2+^ channels as a result of proton‐coupled uptake of di/tripeptides by PepT1 (and sodium‐coupled uptake of free amino acids) or other sodium‐coupled transporters, we inhibited the L‐type Ca^2+^‐channels with nifedipine (10 *μ*mol/L) while stimulating the intestine luminally with peptone. Nifedipine was infused intra‐arterially, beginning 5 min prior to stimulation with peptones, to ensure that the channels were fully inhibited at the time of peptone administration (Fig. 3A).

Nifedipine did not reduce the peptone‐mediated GLP‐1 response when compared with the responses observed in the peptone double‐stimulation control experiment (P2: Fig. 1A; subtracted baseline) (P2: 90 ± 36  fmol/min vs. P2_nif_: 93 ± 64 fmol/min, *P* = 0.9191, *n* = 6) (Fig. 3B). Furthermore, in control experiments, nifedipine did not affect the Gly‐Sar‐mediated GLP‐1 secretion when compared to the second response in a Gly‐Sar double control experiment (G2; Fig. 1C) (baseline‐subtracted values; G2: 57 ± 36 fmol/min vs. G2_nif_:39 ± 24 fmol/min, *P* = 0.4219, *n* = 4–6, Fig. 3C+D), indicating that PepT1 does not mediate GLP‐1 secretion by depolarization‐dependent mechanisms.

To validate that the lack of effect of nifedipine was not related to bio‐inactivity or concentration‐related issues, we tested whether nifedipine inhibited glucose‐stimulated GLP‐1 secretion, which has been demonstrated by several research groups (including our own) to be driven by the opening of L‐type Ca^2+^ channels (secondary to by electrogenic uptake of glucose via sodium‐glucose cotransporter 1 (SGLT1)). Nifedipine (10 *μ*mol/L) strongly inhibited the second glucose‐induced GLP‐1 response (baseline‐subtracted values; Glucose2: 227 ± 40 fmol/min vs. Glucose2_nif_: 91 ± 17 fmol/min, *P* < 0.05, *n* = 4, data not shown).

### Calcium‐Sensing Receptor is located on the basolateral membrane and is involved in peptone‐mediated GLP‐1 secretion

**Figure 4:**
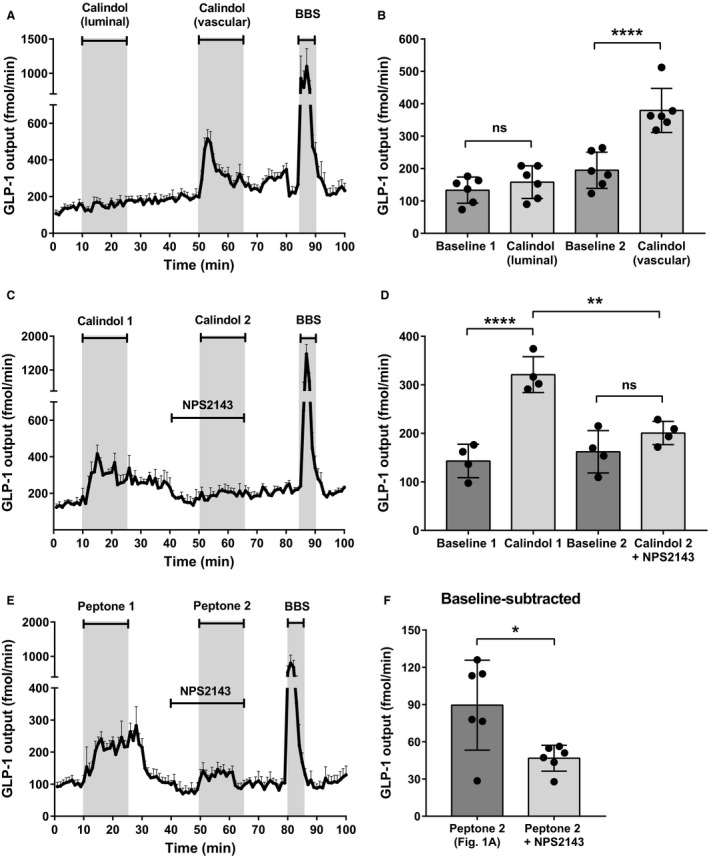
Calcium‐Sensing Receptor is located on the basolateral membrane and is involved in peptone‐mediated GLP‐1 secretion. (A) Total GLP‐1 outputs shown as means ±  SEM. Calindol (50 *μ*mol/L), a positive allosteric modulator of CaSR, was intraluminally infused between minute 11–25 and intravascularly infused between minute 51–65. (B) Mean GLP‐1 outputs at baseline (Baseline 1 and 2) and following stimulation with luminal calindol and vascular calindol. (C) Total GLP‐1 outputs shown as means ± SEM. Calindol (50 *μ*mol/L) was intraluminally infused between minute 11–25 and intravascular infused between minute 51–65. NPS2143 (25 *μ*mol/L), a negative allosteric modulator of CaSR, was intravascularly infused between minute 41–60. (D) Mean GLP‐1 outputs at baseline (Baseline 1 and 2) and following stimulation with vascular calindol (Calindol 1) and calindol + NPS2143 (Calindol 2 + NPS2143). (E) Total GLP‐1 outputs shown as means ± SEM. Peptone (50 mg/mL) was intraluminally infused between minute 11–25 and 51–65. NPS2143 (25 *μ*mol/L), was intravascularly infused between minute 41–60. (F) Mean values of baseline‐subtracted peptone‐stimulated GLP‐1 secretion with and without NPS2143 (Peptone 2; Fig. 1 vs. Peptone 2 + NPS2143). Statistical significance was tested by one‐way ANOVA for repeated measurements followed by Bonferroni post hoc test (Fig. 4B+D) and by unpaired student's *t*‐test (Fig. 4F); NS: nonsignificant: *P* > 0.05. *P < 0.05, **P < 0.01, ***P < 0.001, ****P < 0.0001. *n* = 6 for all groups except Calindol + NPS2143 (Fig. 4C), *n* = 4.

Since our balance of evidence suggested that absorption of protein digestion products is the main driver of peptone‐induced GLP‐1 secretion (Fig. 2), and since peptides are rapidly transported to the vascular side of the gut in the form of amino acids (Fig. 2C), we next investigated the role of a well‐known amino acid sensing receptor, CaSR, in peptone‐stimulated GLP‐1 secretion. To establish whether CaSR played a role for GLP‐1 secretion in general, we initially stimulated the perfused intestine with a specific positive allosteric modulator of CaSR, calindol (50 *μ*mol/L), luminally and vascularly. Calindol strongly stimulated GLP‐1 secretion when infused vascularly (C_vasc_), but had marginal and nonsignificant effects on secretion when administered luminally at the same concentration (C_lum_) (B1: 133 ± 40 fmol/min vs. C_lum_: 158 ± 50 fmol/min, *P* > 0.9999, B2: 195 ± 56 fmol/min vs. C_vasc_: 380 ± 68 fmol/min, *P* < 0.05, *n* = 6) (Fig. 4A+B). Our data therefore suggest that CaSR is located on the basolateral side, and could be involved in the secretory GLP‐1 response to vascular amino acids.

To validate the specificity of calindol as a CaSR agonist, we examined whether we were able to inhibit the vascular response with a negative allosteric modulator of CaSR, NPS2143. NPS2143 (25 *μ*mol/L), inhibited the vascular calindol‐induced (50 *μ*mol/L) GLP‐1 response significantly, supporting the claimed specificity of calindol (B1: 143 ± 35 fmol/min vs. calindol: 321 ± 37 fmol/min, *P* < 0.05, B2: 162 ± 44 fmol/min vs. calindol_+NPS2143_: 201 ± 24 fmol/min, *P* = 0.8946, *n* = 4*,* Fig. 4C+D).

To investigate the involvement of CaSR in the peptone‐stimulated GLP‐1 secretion, we next stimulated the perfused intestine with luminal peptones, while inhibiting CaSR with NPS2143 (25 *μ*mol/L) (Fig. 4E). This inhibited the second peptone‐induced GLP‐1 response (P2: Fig. 1A) significantly, when subtracting baselines (P2: 90 ± 36 fmol/min vs. P2_+NPS2143_: 47 ± 10 fmol/min, *P* < 0.05, *n* = 6) (Fig. 4F), indicating that CaSR is involved in the sensing of absorbed protein digestion products.

## Discussion

GLP‐1 is secreted from L cells in the gastrointestinal tract upon meal intake. The products of digestion contributing to GLP‐1 release include glucose and fructose, fatty acids, and in particular 2‐monoacylglycerols, amino acids, and oligopeptides (Orskov et al. 1991; Tolhurst et al. 2009; Hansen et al. 2012; Kuhre et al. 2014). The mechanisms by which carbohydrates are sensed by the L cells have been relatively well characterized, and include electrogenic sugar (glucose, galactose) uptake by the sodium‐dependent glucose transporter 1 (SGLT1), possibly in conjunction with the metabolism of glucose and closure of ATP‐sensitive K^+^‐channels (Gorboulev et al. 2012; Parker et al. 2012; Röder et al. 2014; Kuhre et al. 2015) but the mechanisms by which ingested fat and protein trigger GLP‐1 release are less well characterized.

**Figure 5:**
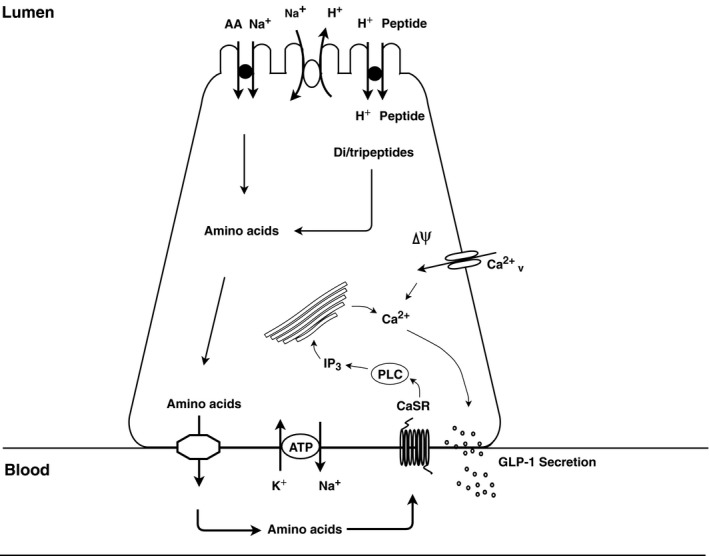
Illustration of the endocrine L cell and the proposed mechanisms by which peptone stimulates GLP‐1 release. Di/tripeptides are taken up by PepT1 and are degraded by cytosolic peptidases to their respective amino acids. In addition, free amino acids present in the intestinal lumen may be taken up by different amino acid transporters. Intracellular amino acids are then transported to the interstitial side through basolateral amino acid transporters, wherefrom they stimulate the L cells by activating amino acid sensors, like CaSR, situated on the basolateral membrane.

In particular, it was not established whether protein and/or amino acids stimulate secretion by activation of luminal or basolateral sensors. As this cannot be investigated in isolated cell systems (because of the inherent loss of cell polarization in cultured cells) and is difficult to investigate in vivo (because control of luminal vs. basolateral exposure is difficult to achieve), we investigated the molecular mechanisms of peptone‐stimulated GLP‐1 secretion using isolated perfused rat small intestines. With this preparation, luminal and vascular exposure to a given stimulus can be strictly controlled and the gut performs in many aspects as it does in vivo: it respires, absorbs nutrients, and has preserved polarization and preserved vascular and nerve supply (Svendsen and Holst 2016; Kuhre et al. 2017), and importantly, it is possible to follow the full dynamic spectrum of secretion, minute for minute. Furthermore, as a consequence of adequate vascular perfusion, both absorptive and secretory processes can proceed without inhibition by accumulating substrates or products. Using this model, we found that peptone‐stimulated GLP‐1 secretion is highly dependent upon absorption of smaller peptides and amino acids in the proximal rat small intestine. Our findings suggest that basolateral sensing of amino acids, produced as a result of intracellular digestion of di/tripeptides, may be the most important mechanism by which L cells sense and react to ingested protein (Fig. 5). This was supported by our finding that amino acids powerfully stimulated GLP‐1 secretion also from the vascular side, and that luminally infused protein digestion products were rapidly absorbed as reflected by a rise in vascular amino acids (Fig. 2C+D). However, the molecular mechanisms underlying amino acid stimulated GLP‐1 secretion are complex and probably dependent on activation of different molecular sensors by the individual amino acid. For instance, glutamine, which has been shown to raise plasma GLP‐1 levels in humans (Greenfield et al. 2009), is thought to stimulate GLP‐1 secretion through activation of two different pathways. One involves membrane depolarization through electrogenic Na^+^‐coupled uptake and another involves elevation of intracellular cAMP levels, the details of which remain unclear (Reimann et al. 2004; Tolhurst et al. 2011). The depolarization would lead to opening of voltage‐gated calcium channels with intracellular calcium increases leading to exocytosis of the hormone. This pathway should be sensitive to blockade of the v‐gated calcium channels with nifedipine. Indeed, this was observed in a study of the effect of peptones on cultured small intestinal epithelial cells (Pais et al. 2016) but in the present study, nifedipine had no effects on peptone‐induced secretion. The difference might be due to different amino acid composition of the peptones applied, but might also derive from the use of a random mixture of isolated cells (which may influence secretion of each other) as opposed to our use of an intact, polarized epithelium. In contrast, the importance of the voltage‐gated calcium channels was clearly illustrated in our experiments by the strong inhibitory effect of nifedipine on glucose‐stimulated GLP‐1 secretion, which clearly involves depolarization by sodium cotransported with glucose. Further experiments with single amino acids thought to be transported together with sodium will hopefully clarify this.

Another sensor that has been suggested to be involved in protein‐stimulated GLP‐1 secretion is CaSR, which is highly expressed in GLP‐1 secreting L cells (Diakogiannaki et al. 2013). Activation of this receptor has been demonstrated to stimulate GLP‐1 secretion in several studies of mixed primary mouse mucosal cell cultures (Diakogiannaki et al. 2013; Pais et al. 2016; Alamshah et al. 2017). CaSR is predominantly G_q_‐coupled, leading to calcium release from intracellular stores (Conigrave and Ward 2013). CaSR is activated by a wide range of L‐amino acids with the aromatic amino acids being the most potent agonists (Conigrave et al. 2000; Conigrave and Hampson 2006). However, structural studies suggest that CaSR is also able to bind oligopeptides (Wang et al. 2006). Importantly, to date, it was unknown whether apical or basolateral activation of CaSR mediates peptone‐stimulated GLP‐1 secretion, as previous studies (in vitro or in vivo) were unable to discriminate between these. Here, we demonstrate that basolateral rather than luminal activation of CaSR mediates the response, since blockage of receptor activity from the vascular side (by NPS2143) inhibited the peptone‐induced secretory response, and since infusion of a CaSR‐specific agonist stimulated GLP‐1 secretion only from the vascular side.

A limitation of our study is that in all our experiments with either peptone or Vamin, the second GLP‐1 response was lower than the first albeit the amount of absorbed amino acids was similar during both stimulations. The relatively short washing period in between the two stimulations (20 min) could explain this, as the molecular sensors involved may have been saturated/desensitized by the first stimulation by the time of the second stimulation. More specifically, the CaSR may have been desensitized or internalized during/after the first stimulation. Supporting this, two similar GLP‐1 responses were observed in a glucose double control experiment (Glu1: 487 ± 77 fmol/min vs. Glu2: 434 ± 57 fmol/min, *P* > 0.9999, *n* = 4, data not shown), demonstrating that the lower second GLP‐1 response seen is not due to a general reduced viability of the organ by the time of the second stimulation period but instead seems to depend on the stimulatory compound and the signaling pathway involved. This could likewise explain the lower GLP‐1 response seen after infusion of vascular amino acids compared to infusion of luminal amino acids (Fig. 1E+F), as the amino acid sensors may have been internalized/desensitized after the first stimulation period with luminally infused amino acids. Moreover, the PepT1 blocker used in this study (4‐AMBA) appeared not to be specific for di/tripeptide uptake but also blocked the uptake of free amino acids in the intestinal lumen. Although we were not able to distinguish between the uptake of free amino acids and di/tripeptides, when stimulating with peptone, our study clearly demonstrated that absorption of both digestion products is essential for protein‐stimulated GLP‐1 secretion in the proximal rat small intestine, since addition of 4‐AMBA eliminated the peptone‐induced GLP‐1 response.

The contribution of individual amino acids to total peptone‐stimulated GLP‐1 secretion, and the respective molecular pathways involved, awaits further investigation. Key sensors to be investigated are: GPRC6A, T1R1/T1R3, GPR142, GPR93, and GPR35, although the importance of GPRC6A was recently questioned (Clemmensen et al. 2017). In addition, how Gly‐Sar stimulates GLP‐1 secretion deserves further exploration, as the mechanism is currently unclear. It is possible that Gly‐Sar stimulate sensors on the basolateral side as a dipeptide, similar to amino acids.

In conclusion, our data indicated that proteins stimulate GLP‐1 secretion through absorbed amino acids, which activate amino acid sensing receptors, including CaSR, situated on the basolateral membrane (Fig. 5). As mentioned in the introduction, therapies based on stimulation of the endogenous secretion of L‐cell products (as seen after gastric bypass operations) might be beneficial compared to the administration of exogenous hormones, and for this a detailed knowledge of the stimulatory mechanisms is essential. In this respect, stimulation with proteins is of particular interest in view of their robust effect on secretion and appetite (Elliott et al. 1993; Weigle et al. 2005; Belza et al. 2013; van der Klaauw et al. 2013). Currently, the effects of oral amino acid supplementation on GLP‐1 secretion in humans have been variable (Nilsson et al. 2007; Greenfield et al. 2009; Lindgren et al. 2015; Tricò et al. 2018), and therefore warrants further clarification. The essential CaSR is expressed in many tissues of the body (kidneys, gut, parathyroid gland) and agonism may therefore have many effects, but oral administration of absorbable agonists might preferentially stimulate intestinal receptors. The robust stimulation of GLP‐1 secretion observed in the current experiments supports further investigations into protein‐induced secretion.

## Conflict of Interest

The authors of this work declare no potential conflicts of interest relevant to this article.

## References

[phy214056-bib-0001] Alamshah, A. , E. Spreckley , M. Norton , J. S. Kinsey‐Jones , A. Amin , A. Ramgulam , et al. 2017 l‐phenylalanine modulates gut hormone release and glucose tolerance, and suppresses food intake through the calcium‐sensing receptor in rodents. Int. J. Obes. 41:1693–1701. 10.1038/ijo.2017.164 PMC5678004 28792489

[phy214056-bib-0002] Baggio, L. L. , and D. J. Drucker . 2007 Biology of incretins: GLP‐1 and GIP. Gastroenterology 132:2131–2157. 17498508 10.1053/j.gastro.2007.03.054

[phy214056-bib-0003] Belza, A. , C. Ritz , M. Q. Sørensen , J. J. Holst , J. F. Rehfeld , and A. Astrup . 2013 Contribution of gastroenteropancreatic appetite hormones to protein‐induced satiety. Am. J. Clin. Nutr. 97:980–989. 23466396 10.3945/ajcn.112.047563

[phy214056-bib-0004] Clemmensen, C. , C. V. Jørgensen , S. Smajilovic , and H. Bräuner‐Osborne . 2017 Robust GLP‐1 secretion by basic L‐amino acids does not require the GPRC6A receptor. Diabetes Obes. Metab. 19:599–603. 27943578 10.1111/dom.12845

[phy214056-bib-0005] Conigrave, A. D. , and D. R. Hampson . 2006 Broad‐spectrum l‐amino acid sensing by class 3 G‐protein‐coupled receptors. Trends Endocrinol. Metab. 17:398–407. 17085057 10.1016/j.tem.2006.10.012

[phy214056-bib-0006] Conigrave, A. D. , and D. T. Ward . 2013 Calcium‐sensing receptor (CaSR): pharmacological properties and signaling pathways. Best Pract. Res. Clin. Endocrinol. Metab. 27:315–331. 23856262 10.1016/j.beem.2013.05.010

[phy214056-bib-0007] Conigrave, A. D. , S. J. Quinn , and E. M. Brown . 2000 L‐amino acid sensing by the extracellular Ca2^+^‐sensing receptor. Proc. Natl Acad. Sci. USA 97:4814–4819. 10781086 10.1073/pnas.97.9.4814 PMC18315

[phy214056-bib-0008] Diakogiannaki, E. , R. Pais , G. Tolhurst , H. E. Parker , J. Horscroft , B. Rauscher , et al. 2013 Oligopeptides stimulate glucagon‐like peptide‐1 secretion in mice through proton‐coupled uptake and the calcium‐sensing receptor. Diabetologia 56:2688–2696. 24045836 10.1007/s00125-013-3037-3 PMC3825574

[phy214056-bib-0009] Dranse, H. J. , T. M. Z. Waise , S. C. Hamr , P. V. Bauer , M. A. Abraham , B. A. Rasmussen , et al. 2018 Physiological and therapeutic regulation of glucose homeostasis by upper small intestinal PepT1‐mediated protein sensing. Nat. Commun. 9:1118. 29549253 10.1038/s41467-018-03490-8 PMC5856761

[phy214056-bib-0010] Drucker, D. J. , and M. A. Nauck . 2006 The incretin system: glucagon‐like peptide‐1 receptor agonists and dipeptidyl peptidase‐4 inhibitors in type 2 diabetes. Lancet 368:1696–1705. 17098089 10.1016/S0140-6736(06)69705-5

[phy214056-bib-0011] Elliott, R. M. , L. M. Morgan , J. A. Tredger , S. Deacon , J. Wright , and V. Marks . 1993 Glucagon‐like peptide‐1 (7‐36)amide and glucose‐dependent insulinotropic polypeptide secretion in response to nutrient ingestion in man: acute post‐prandial and 24‐h secretion patterns. J. Endocrinol. 138:159–166. 7852887 10.1677/joe.0.1380159

[phy214056-bib-0012] Furness, J. B. , W. A. Kunze , and N. Clerc . 1999 Nutrient tasting and signaling mechanisms in the gut. II. The intestine as a sensory organ: neural, endocrine, and immune responses. Am. J. Physiol. 277:G922–G928. 10564096 10.1152/ajpgi.1999.277.5.G922

[phy214056-bib-0013] Gorboulev, V. , A. Schürmann , V. Vallon , H. Kipp , A. Jaschke , D. Klessen , et al. 2012 Na(+)‐D‐glucose cotransporter SGLT1 is pivotal for intestinal glucose absorption and glucose‐dependent incretin secretion. Diabetes 61:187–196. 22124465 10.2337/db11-1029 PMC3237647

[phy214056-bib-0014] Greenfield, J. R. , I. S. Farooqi , J. M. Keogh , E. Henning , A. M. Habib , A. Blackwood , et al. 2009 Oral glutamine increases circulating glucagon‐like peptide 1, glucagon, and insulin concentrations in lean, obese, and type 2 diabetic subjects. Am. J. Clin. Nutr. 89:106–113. 19056578 10.3945/ajcn.2008.26362 PMC4340573

[phy214056-bib-0015] Hall, W. L. , D. J. Millward , S. J. Long , and L. M. Morgan . 2003 Casein and whey exert different effects on plasma amino acid profiles, gastrointestinal hormone secretion and appetite. Br. J. Nutr. 89:239–248. 12575908 10.1079/BJN2002760

[phy214056-bib-0016] Hansen, H. S. , M. M. Rosenkilde , J. J. Holst , and T. W. Schwartz . 2012 GPR119 as a fat sensor. Trends Pharmacol. Sci. 33:374–381. 22560300 10.1016/j.tips.2012.03.014

[phy214056-bib-0017] Herrmann, C. , R. Göke , G. Richter , H. C. Fehmann , R. Arnold , and B. Göke . 1995 Glucagon‐like peptide‐1 and glucose‐dependent insulin‐releasing polypeptide plasma levels in response to nutrients. Digestion 56:117–126. 7750665 10.1159/000201231

[phy214056-bib-0018] Holst, J. J. 2007 The physiology of glucagon‐like peptide 1. Physiol. Rev. 87:1409–1439. 17928588 10.1152/physrev.00034.2006

[phy214056-bib-0019] Holst, J. J. , and C. F. Deacon . 2005 Glucagon‐like peptide‐1 mediates the therapeutic actions of DPP‐IV inhibitors. Diabetologia 48:612–615. 15759106 10.1007/s00125-005-1705-7

[phy214056-bib-0020] van der Klaauw, A. A. , J. M. Keogh , E. Henning , V. M. Trowse , W. S. Dhillo , M. A. Ghatei , et al. 2013 High protein intake stimulates postprandial GLP1 and PYY release. Obesity 21:1602–1607. 23666746 10.1002/oby.20154 PMC6548554

[phy214056-bib-0021] Kuhre, R. E. , F. M. Gribble , B. Hartmann , F. Reimann , J. A. Windeløv , J. F. Rehfeld , et al. 2014 Fructose stimulates GLP‐1 but not GIP secretion in mice, rats, and humans. Am. J. Physiol. Gastrointest. Liver Physiol. 306:G622–G630. 24525020 10.1152/ajpgi.00372.2013 PMC3962593

[phy214056-bib-0022] Kuhre, R. E. , C. R. Frost , B. Svendsen , and J. J. Holst . 2015 Molecular mechanisms of glucose‐stimulated GLP‐1 secretion from perfused rat small intestine. Diabetes 64:370–382. 25157092 10.2337/db14-0807

[phy214056-bib-0023] Kuhre, R. E. , C. B. Christiansen , M. Y. Saltiel , N. J. Wewer Albrechtsen , and J. J. Holst . 2017 On the relationship between glucose absorption and glucose‐stimulated secretion of GLP‐1, neurotensin, and PYY from different intestinal segments in the rat. Physiol. Rep. 5:23. 10.14814/phy2.13507 PMC5727272 29199179

[phy214056-bib-0024] Lindgren, O. , G. Pacini , A. Tura , J. J. Holst , C. F. Deacon , and B. Ahrén . 2015 Incretin effect after oral amino acid ingestion in humans. J. Clin. Endocrinol. Metab. 100:1172–1176. 25490278 10.1210/jc.2014-3865

[phy214056-bib-0025] Mace, O. J. , M. Schindler , and S. Patel . 2012 The regulation of K‐ and L‐cell activity by GLUT2 and the calcium‐sensing receptor CasR in rat small intestine. J. Physiol. 590:2917–2936. 22495587 10.1113/jphysiol.2011.223800 PMC3448156

[phy214056-bib-0026] Matsumura, K. , T. Miki , T. Jhomori , T. Gonoi , and S. Seino . 2005 Possible role of PEPT1 in gastrointestinal hormone secretion. Biochem. Biophys. Res. Comm. 336:1028–1032. 16181611 10.1016/j.bbrc.2005.08.259

[phy214056-bib-0027] Nilsson, M. , J. J. Holst , and I. M. Björck . 2007 Metabolic effects of amino acid mixtures and whey protein in healthy subjects: studies using glucose‐equivalent drinks. Am. J. Clin. Nutr. 85:996–1004. 17413098 10.1093/ajcn/85.4.996

[phy214056-bib-0028] Orskov, C. , J. Jeppesen , S. Madsbad , and J. J. Holst . 1991 Proglucagon products in plasma of noninsulin‐dependent diabetics and nondiabetic controls in the fasting state and after oral glucose and intravenous arginine. J. Clin. Investig. 87:415–423. 1991827 10.1172/JCI115012 PMC295092

[phy214056-bib-0029] Pais, R. , F. M. Gribble , and F. Reimann . 2016 Signalling pathways involved in the detection of peptones by murine small intestinal enteroendocrine L‐cells. Peptides 77:9–15. 26215048 10.1016/j.peptides.2015.07.019 PMC4788506

[phy214056-bib-0030] Parker, H. E. , A. Adriaenssens , G. Rogers , P. Richards , H. Koepsell , F. Reimann , et al. 2012 Predominant role of active versus facilitative glucose transport for glucagon‐like peptide‐1 secretion. Diabetologia 55:2445–2455. 22638549 10.1007/s00125-012-2585-2 PMC3411305

[phy214056-bib-0031] Pi‐Sunyer, X. , A. Astrup , K. Fujioka , F. Greenway , A. Halpern , M. Krempf , et al. 2015 A randomized, controlled trial of 3.0 mg of liraglutide in weight management. N. Engl. J. Med. 373:11–22. 26132939 10.1056/NEJMoa1411892

[phy214056-bib-0032] Raben, A. , L. Agerholm‐Larsen , A. Flint , J. J. Holst , and A. Astrup . 2003 Meals with similar energy densities but rich in protein, fat, carbohydrate, or alcohol have different effects on energy expenditure and substrate metabolism but not on appetite and energy intake. Am. J. Clin. Nutr. 77:91–100. 12499328 10.1093/ajcn/77.1.91

[phy214056-bib-0033] Reimann, F. , L. Williams , Xavier G. da Silva , G. A. Rutter , and F. M. Gribble . 2004 Glutamine potently stimulates glucagon‐like peptide‐1 secretion from GLUTag cells. Diabetologia 47:1592–1601. 15365617 10.1007/s00125-004-1498-0

[phy214056-bib-0034] Röder, P. V. , K. E. Geillinger , T. S. Zietek , B. Thorens , H. Koepsell , and H. Daniel . 2014 The role of SGLT1 and GLUT2 in intestinal glucose transport and sensing. PLoS ONE 9:e89977. 24587162 10.1371/journal.pone.0089977 PMC3935955

[phy214056-bib-0035] Steinert, R. E. 2011 Nutrient sensing in the gut: interactions between chemosensory cells, visceral afferents and the secretion of satiation peptides. Physiol. Behav. 105:62–70. 21376067 10.1016/j.physbeh.2011.02.039

[phy214056-bib-0036] Svendsen, B. , and J. J. Holst . 2016 Regulation of gut hormone secretion. Studies using isolated perfused intestines. Peptides 77:47–53. 26275337 10.1016/j.peptides.2015.08.001

[phy214056-bib-0037] Svendsen, B. , J. Pedersen , N. J. W. Albrechtsen , B. Hartmann , S. Toräng , J. F. Rehfeld , et al. 2015 An analysis of cosecretion and coexpression of gut hormones from male rat proximal and distal small intestine. Endocrinology 156:847–857. 25535831 10.1210/en.2014-1710

[phy214056-bib-0038] Tolhurst, G. , F. Reimann , and F. M. Gribble . 2009 Nutritional regulation of glucagon‐like peptide‐1 secretion. J. Physiol. 587:27–32. 19001044 10.1113/jphysiol.2008.164012 PMC2670019

[phy214056-bib-0039] Tolhurst, G. , Y. Zheng , H. E. Parker , A. M. Habib , F. Reimann , and F. M. Gribble . 2011 Glutamine triggers and potentiates glucagon‐like peptide‐1 secretion by raising cytosolic Ca ^2+^ and cAMP. Endocrinology 152:405–413. 21209017 10.1210/en.2010-0956 PMC3140224

[phy214056-bib-0040] Tricò, D. , S. Frascerra , S. Baldi , A. Mengozzi , L. Nesti , A. Mari , et al. 2018 The insulinotropic effect of a high‐protein nutrient preload is mediated by the increase of plasma amino acids in type 2 diabetes. Eur. J. Nutr. 2018:1–9 10.1007/s00394-018-1778-y. 30008106

[phy214056-bib-0041] Wang, M. , Y. Yao , D. Kuang , and D. R. Hampson . 2006 Activation of family C G‐protein‐coupled receptors by the tripeptide glutathione. J. Biol. Chem. 281:8864–8870. 16455645 10.1074/jbc.M512865200

[phy214056-bib-0042] Weigle, D. S. , P. A. Breen , C. C. Matthys , H. S. Callahan , K. E. Meeuws , V. R. Burden , et al. 2005 A high‐protein diet induces sustained reductions in appetite, ad libitum caloric intake, and body weight despite compensatory changes in diurnal plasma leptin and ghrelin concentrations. Am. J. Clin. Nutr. 82:41–48. 16002798 10.1093/ajcn.82.1.41

[phy214056-bib-0043] Zander, M. , S. Madsbad , J. L. Madsen , and J. J. Holst . 2002 Effect of 6‐week course of glucagon‐like peptide 1 on glycaemic control, insulin sensitivity, and beta‐cell function in type 2 diabetes: a parallel‐group study. Lancet 359:824–830. 11897280 10.1016/S0140-6736(02)07952-7

